# Editorial: Oesophageal Atresia-Tracheoesophageal Fistula

**DOI:** 10.3389/fped.2017.00190

**Published:** 2017-08-31

**Authors:** Usha Krishnan, Christophe Faure

**Affiliations:** ^1^Department of Pediatric Gastroenterology, Sydney Children’s Hospital, Sydney, NSW, Australia; ^2^Discipline of Pediatrics, School of Women’s and Children’s Health, University of New South Wales, Sydney, NSW, Australia; ^3^Division of Pediatric Gastroenterology, Sainte-Justine Hospital, Montreal, QC, Canada; ^4^Department of Pediatrics, Université de Montréal, Montreal, QC, Canada

**Keywords:** esophageal atresia, pediatric surgery, fistula, carcinoma, gastroenterology

**Editorial on the Research Topic**

**Oesophageal Atresia-Tracheoesophageal Fistula**

Esophageal atresia is among the most common congenital digestive malformations, affecting 1 in 3,000 newborn babies at birth. Since the first successful primary repair of esophageal atresia (EA) in 1941, improvements in operative and perioperative care have led to better outcomes, and thus we have seen an evolvement from mortality to morbidity and quality-of-life issues. In fact, EA is no longer a mere neonatal surgical problem but rather a lifelong problem for the patient. It appears that respiratory, nutritional, and gastroenterological issues are the most prevalent sequelae—not only in the first years of life but also in adolescence and adulthood. Hence a multidisciplinary approach has been advocated by many centers in order to coordinate and optimize the management of these patients at all stages of life. In 2010, the First International Workshop on Esophageal Atresia was held in Lille, France. The success of that event established a new model of conference based not on medical subspecialty but specifically on disease, bringing together diverse disciplines all linked together by their common interest and expertise in treating EA. The International Network of Esophageal Atresia (INoEA) was founded in 2013. *On September 15–16, 2016, the fourth International Conference on Esophageal Atresia “Coming Together” took place in* Sydney, NSW, Australia. More than 200 participants from all over the world attended the conference. There were over 80 scientific abstracts submitted. All the categories of people involved in the care of EA patients were represented; not only neonatologists, pediatricians, surgeons, gastroenterologists, otolaryngologists, pulmonologists, radiologists, anesthesiologists, intensivists, but also, nurses, dieticians, speech pathologists, psychologists, occupational therapists, social workers, parents of patients, and children and adults with EA. The scientific program was both comprehensive and innovative, covering the entire spectrum of disease from genetic predisposition and pathophysiology, aspiration risk and chronic respiratory morbidity (CRM), investigation and management of gastroesophageal reflux disease including risk of Barrett’s and esophageal cancer, tracheomalacia, and its management including novel techniques like posterior aortopexy, newer techniques of reflux and motility testing, techniques of surgical repair and role of fundoplication, stricture management, feeding difficulties and their practical management, quality of life of these patients, the need for ongoing care with transition to adulthood, and the need for an international registry. For the first time, the ESPGHAN–NASPGHAN consensus guidelines on the management of gastrointestinal complication in children with EA were presented and very well received not only by the clinicians involved in the care of these patients but also the parent support groups. Innovative topics, which were presented for the first time and which stimulated interesting discussions included: deliberations from the InoEA working group on Long Gap Esophageal Atresia, Preliminary deliberations from the respiratory working group, Prevalence of abnormal Gastric Function and Dumping syndrome in this cohort, the role of pH-impedance testing for GERD and high-resolution impedance manometry for pharyngoesophageal function testing, a validated quality of life score and academic performance in EA patients, molecular profiling of EA patients with eosinophilic esophagitis (EoE), the Sydney experience with the Foker technique, risks associated with general anesthetic and radiation exposure and role of stem cell therapy and neo esophagus in the future. Experts from India, China, and Japan spoke for the first time about management of EA in their countries. The talks from a teenager and adult EA survivors were especially inspiring for the audience. The success of the event confirmed the importance of adopting a multidisciplinary approach and creating links between not only pediatric and adult medicine but also with parent support groups.

This special edition of “Frontiers in Pediatrics” contains summaries and review articles of selected presentations delivered by the distinguished guest speakers during the conference.

The comprehensive review by Kovesi on “*Aspiration risk and respiratory complications in patients with esophageal atresia*” summarizes current knowledge on the degree to which aspiration is responsible for CRM in this cohort. While the etiology of aspiration is multifactorial, diagnosing aspiration remains medically challenging.

The paper by Bergeron et al. looks at the “*Management of cyanotic spells in children with oesophageal atresia*.” In concordance with the recently published ESPGHAN–NASPGHAN Guidelines on the management of gastrointestinal complications in children with EA/tracheoesophageal fistula (TEF), this article highlights the importance of a multidisciplinary diagnostic evaluation of cyanotic spells prior to surgical intervention with aortopexy and or fundoplication (1).

The paper by Faure and Grunder looks at “*Dysmotility in esophageal atresia: pathophysiology, characterisation and treatment*.”

Anastomotic stricture (AS) is the most common complication following operative repair of EA, and this is looked at in the comprehensive review article “*Anastomotic Strictures after esophageal Atresia Repair: incidence, investigations, and Management, including Treatment of Refractory and Recurrent Strictures*” by Tambucci et al. Since AS formation is likely influenced by GER, and the ESPGHAN–NASPGHAN Guidelines suggest a systematic routine treatment with PPI for 1 year after surgical correction, including in asymptomatic patients (1); it will be interesting to investigate whether this routine practice will decrease the stricture formation in the future. The Guidelines also state that “Eosinophilic esophagitis (EoE) needs to be excluded in EA patients of all ages with dysphagia, reflux symptoms, coughing, choking, or recurrent strictures that are refractory to PPI” (1), and prospective studies will help delineate the true incidence of EoE in EA patients with recurrent strictures.

Feeding difficulties are common in patients with repaired EA, and this review by Mahoney and Rosen on “*Feeding problems and their underlying mechanisms in esophageal atresia-tracheoesophageal fistula patient*” highlights multifactorial etiology for abnormal feeding in this cohort.

The article by Rintala looks at “*Fundoplication in patients with esophageal atresia: patient selection, indications, and outcomes*.” Fundoplication is frequently required in EA patients; however, the indications for fundoplication are often not scientifically delineated. The recent ESPGHAN–NASPGHAN guidelines list refractory anastomotic stenosis, long-gap EA, poorly controlled GERD despite maximal medical therapy, long-term dependency on transpyloric feeding, and cyanotic spells as indications to consider anti-reflux surgery in children with EA (1). The guidelines also state that EoE needs to be excluded in EA patients of all ages with dysphagia, reflux symptoms, coughing, choking, or recurrent strictures that are refractory to PPI; and other abnormalities like laryngeal cleft, vocal cord paralysis, missed or recurrent fistula, AS, congenital stenosis, and vascular ring should be ruled out in EA patients with respiratory symptoms before proceeding to anti-reflux surgery (1).

In the EA spectrum, long-gap esophageal atresia (LGEA) is only a small portion (10%), but the inability to perform a primary esophageal anastomosis poses additional challenges. The position paper by Van Der Zee et al. on “*Position paper of INoEA Working Group on Long Gap Esophageal Atresia (LGEA)*,” after review of the literature and expert discussion concluded that LGEA should be defined as any EA that has no intra-abdominal air, realizing that this defines EA with no distal tracheoesophageal fistula (TEF). In light of the infrequent occurrence of LGEA and the technically demanding techniques involved to achieve esophageal continuity, the working group strongly advised to develop centers of expertise for the management and follow-up of these very complex patients.

Esophageal atresia patients are predisposed to gastroesophageal reflux as a result of the altered esophageal anatomy and motility. The article *on* “*Impedance testing in esophageal atresia patients*” by Hassan and Mousa looks at the role of multichannel intraluminal impedance testing in the investigation and treatment of GERD in this population.

The article on “*Recent advances in motility testing in patients with esophageal atresia*” by Rommel et al. looks at the recent developments in this field. The authors elegantly describe how high-resolution manometry combined with impedance measurements characterizes the interplay between esophageal motor function and bolus clearance. The authors use a novel pressure flow analysis method as an integrated analysis method of manometric and impedance measurements, to differentiate patients with impaired esophagogastric junction (relaxation) from patients with bolus outflow disorders.

While much is known about the abnormal esophageal function and poor motility in EA–TEF patients, little is known about gastric function in this cohort. The review by Duvoisin and Krishnan on “*Gastric function in children with esophageal atresia-tracheosophageal fistula*” gives us a comprehensive understanding of gastric function and potential treatment modalities in EA–TEF patients with abnormal gastric function.

The management of EA remains challenging. This article by Perin et al. on “*An update on foregut molecular embryology and the role of regenerative medicine therapies*” outlines the most current understanding of the molecular embryology underlying foregut development and EA, and also explores the promise of regenerative medicine.

Data on EA prevalence, management, and long-term outcome are lacking because the available data come from small retrospective series from tertiary referral centers. The article on “*The importance of an international registry for and collaborative research on esophageal atresia*” by Gottrand et al. describes how an international multicenter registry would provide strong epidemiological data from large population-based cohorts on EA prevalence, incidence, treatment, long-term morbidity, and prognosis and thereby provide accurate data for evaluation of the current guidelines for EA management.

We look forward to the next fifth international conference on EA in Rome in 2019 where we are sure that many new advancements and innovations in the field will be presented.

## Author Contributions

All authors listed have made a substantial, direct, and intellectual contribution to the work and approved it for publication.

## Conflict of Interest Statement

The authors declare that the research was conducted in the absence of any commercial or financial relationships that could be construed as a potential conflict of interest.
